# Supplementary material for: SARS-CoV-2 Spike Does Not Possess Intrinsic Superantigen-like Inflammatory Activity
Source: Cells. 2022 Aug 15;11(16):2526. doi: 10.3390/cells11162526 (PMC9406418; doi:10.3390/cells11162526)
Supplement: Supplementary file 1 [file cells-11-02526-s001.zip › cells-1872336-supplementary.pdf]

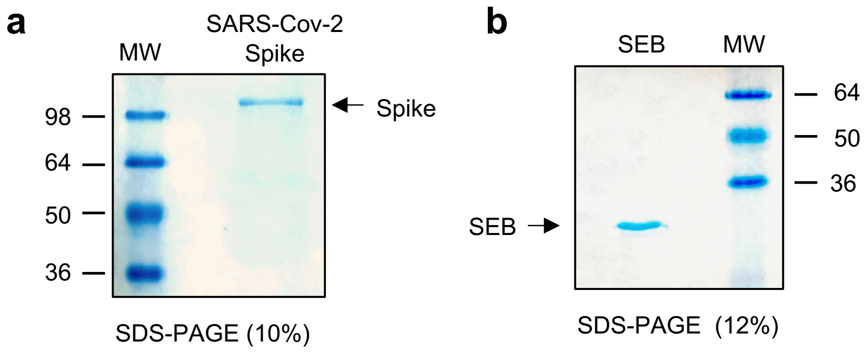

Figure S1: Analysis of recombinant SARS-CoV-2 Spike and SEB proteins. (**a, b**) The purity and integrity of recombinant SARS-CoV-2 Spike (**a**) and SEB (**b**) were analysed by SDS-PAGE and Coomassie blue staining. The positions of Spike, SEB and the molecular weight (MW), expressed in kDa, are indicated.

a

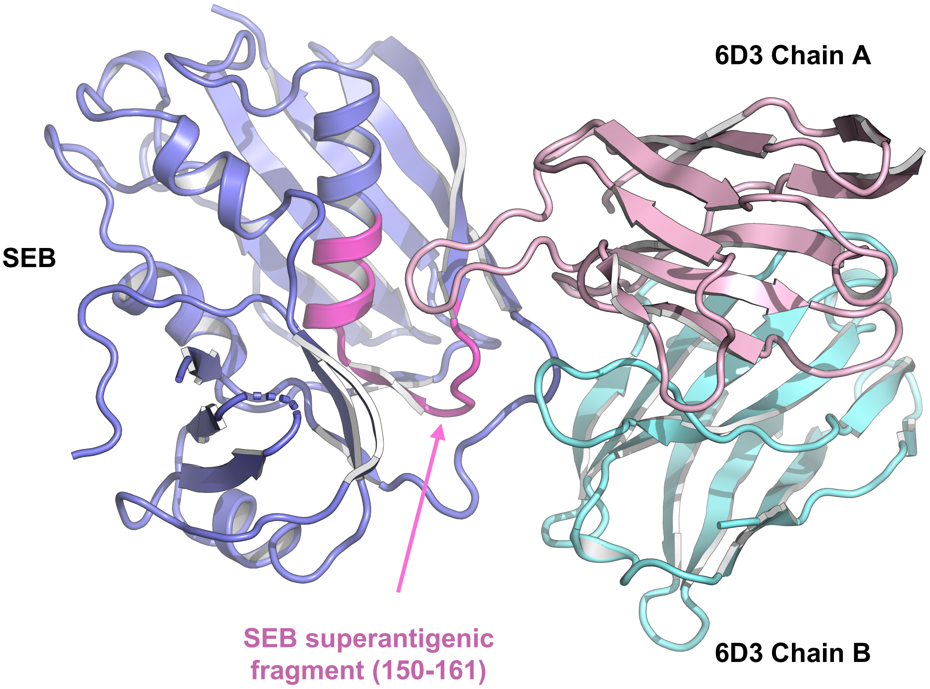

b

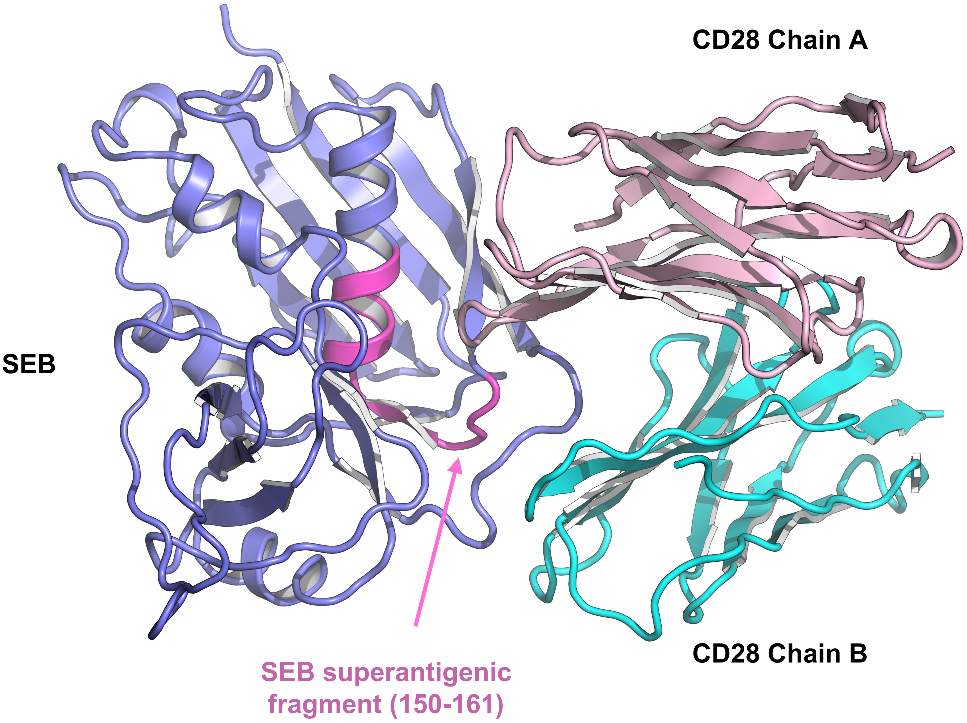

Figure S2. Comparison of SEB bound to 6D3 Ab and CD28. (a) Crystal Structure of SEB (slate) bound to a neutralizing Ab, 6D3 (pink and cyan; residues 1-220) (PDB: 4RGN). The SEB superantigenic fragment (residues 150-161) making contacts with 6D3 is highlighted in violet. (**b)** Docked model of the crystal structure of human CD28 (pink and cyan; PDB: 1YJD) to SEB (PDB: 4RGN). The docking model was obtained by initially superposing each chain of CD28 to 6D3 (RMSD: 2.8 Å) in the structure with PDB 4RGN, and then energy minimizing the whole complex.

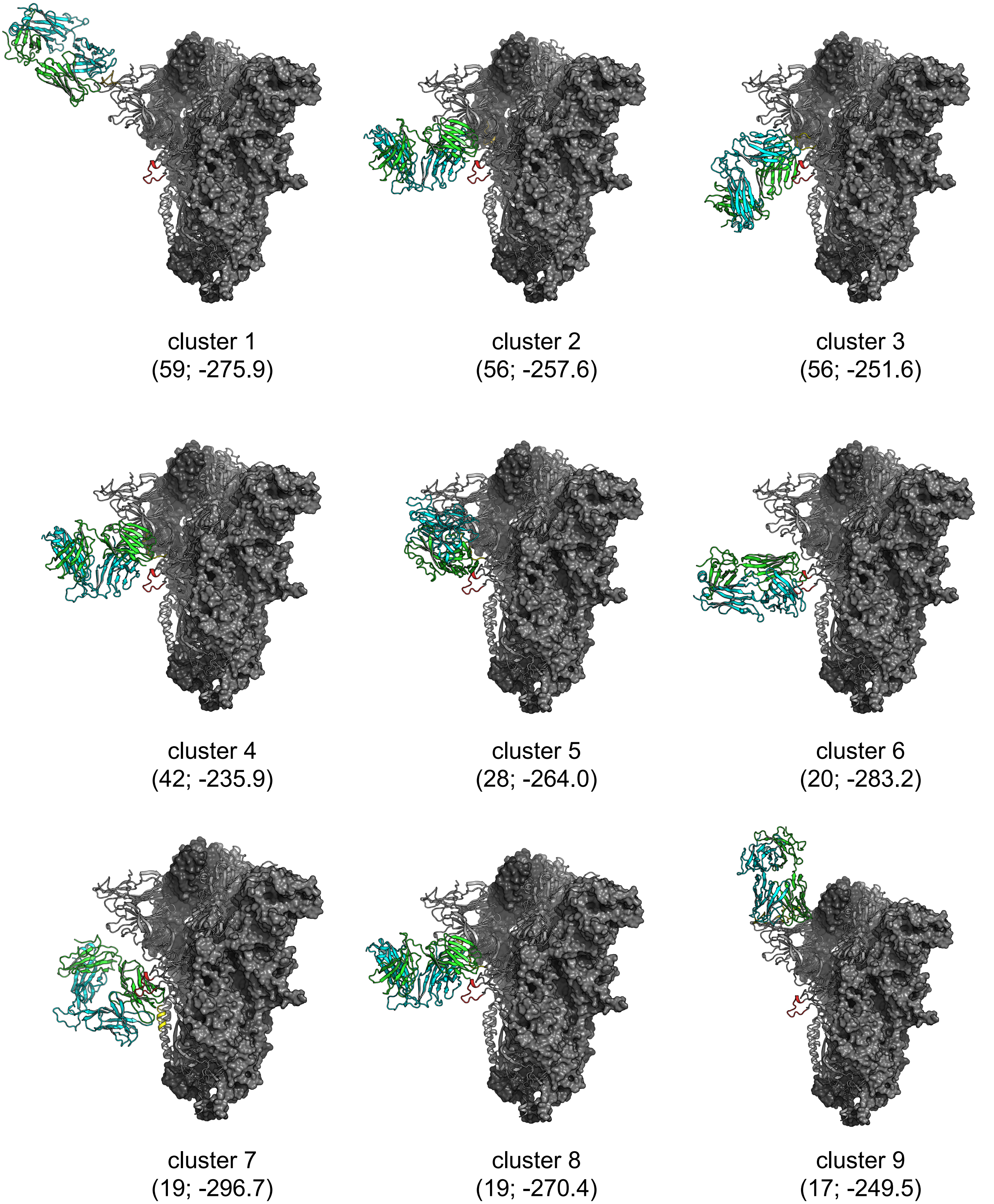

Figure S3. Top-2-10 ranking models of interaction between SARS-CoV-2 spike and αβTCR, as predicted by ClusPro 2.0. The SARS-CoV-2 spike trimer is shown in grey, with the interacting chain as cartoons and the others as surface. The αβTCR structure (PDB: 2XN9) is represented as green and cyan cartoons. The PRRARS insert of the spike protein is coloured in red. The epitope that is recognized is coloured in yellow. The number of members for each cluster and the mean energy is shown in parenthesis. Only in one case (Cluster 6; 20 members; Mean Energy of -283.2) the spike binding epitope contained the PRRA insert.

Table S1. Results of Protein Docking between SARS-CoV-2 spike and TCR TRAV27/TRBV19.

Cluster	Members	Representative	Weighted Score
0	110	Center	-267.4
		Lowest Energy	-298.7
1	59	Center	-275.9
		Lowest Energy	-275.9
2	56	Center	-257.6
		Lowest Energy	-281.3
3	56	Center	-251.6
		Lowest Energy	-301.7
4	42	Center	-235.9
		Lowest Energy	-272.8
5	28	Center	-264.0
		Lowest Energy	-283.5
6	20	Center	-283.2
		Lowest Energy	-283.2
7	19	Center	-296.7
		Lowest Energy	-296.7
8	19	Center	-270.4
		Lowest Energy	-270.4
9	17	Center	-249.5
		Lowest Energy	-249.5
10	17	Center	-234.5
		Lowest Energy	-283.3
11	16	Center	-254.2
		Lowest Energy	-296.2
12	16	Center	-257.7
		Lowest Energy	-257.7
13	15	Center	-279.8
		Lowest Energy	-279.8
14	14	Center	-270.2
		Lowest Energy	-270.2
15	13	Center	-258.1
		Lowest Energy	-258.1
16	13	Center	-249.4
		Lowest Energy	-250.9
17	13	Center	-243.3
		Lowest Energy	-251.5
18	12	Center	-258.2
		Lowest Energy	-258.2
19	11	Center	-237.9
		Lowest Energy	-242.3
20	10	Center	-273.0
		Lowest Energy	-273.0
21	10	Center	-261.6
		Lowest Energy	-261.6
22	5	Center	-233.8
		Lowest Energy	-270.0
23	3	Center	-241.8
		Lowest Energy	-241.8
